# Personalia

**Published:** 1913-12-06

**Authors:** 


### Personalia.

Mr. Henry O'Hara, a Melbourne surgeon, has left Australia for England. He is proceeding to the London Radium Institute to acquaint himself with the latest methods of treatment by radium. Mr. O'Hara will return to Melbourne in January.

Major R. J. Blackham, honorary surgeon to the Viceroy, who is the Assistant Commissioner of the St. John Ambulance Brigade in India, was the recipient recently of an address of congratulation from the Bombay Corps of the Brigade on being created C.I.E. The address was enclosed in a silver casket.

At a quarterly Court of Governors of the Royal Victoria Infirmary, Newcastle-on-Tyne, held on November 27, Mr. James Rutherford Morison, M.A., M.D., F.R.C.S., in recognition of his distinguished services to the institution, was appointed a consulting surgeon. At the same meeting an illuminated address was presented to Mr. Morison on behalf of the Governors. Mr. James Rutherford Morison, in accordance with the sixty years' age limit, retired from his full surgeonship on October 10, 1913. No surgeon in the North of England is better known or has done more for his profession, and all will learn with regret that one, to all appearances as strong and active as ever, should be compelled to relinquish his office. In returning thanks to the Governors, Mr. Morison made an interesting comparison between hospital surgery twenty-five years ago and hospital surgery at the present day. His speech was an optimistic and refreshing one.

### HOSPITAL OFFICERS' ANNUAL DINNER.

The eleventh annual dinner of the Incorporated Association of Hospital Officers will be held at the Holborn Restaurant on Wednesday, December 10, at seven o'clock. The Right Hon. Lord Justice Kennedy will be the guest of the evening.

